# Macrophages: key conductors behind perivascular inflammation and vascular remodeling in hypoxia-induced pulmonary hypertension

**DOI:** 10.1172/JCI190957

**Published:** 2025-03-17

**Authors:** Edda Spiekerkoetter

**Affiliations:** Division of Pulmonary, Allergy and Critical Care Medicine, Vera Moulton Wall Center for Pulmonary Vascular Disease, Department of Medicine, Stanford University School of Medicine, Stanford, California, USA.

## Abstract

Pulmonary hypertension (PH) encompasses a heterogenous group of disorders with the common feature of increased pulmonary arterial pressures. Patients with PH associated with lung disease and/or hypoxia undergo immune-mediated vascular remodeling that includes thickening of the muscular layer surrounding arteries and arterioles. In this issue of the JCI, Kumar and colleagues examined the role of interstitial macrophages in a model of high-altitude PH. Resident interstitial macrophages increased, proliferated, and expressed CCL2, a monocyte chemoattractant ligand. There was also a rise in CCR2^+^ macrophages expressing thrombospondin-1, which is known to activate vascular remodeling through TGF-β. Blocking monocyte recruitment partially reduced hypoxic PH, and corticosteroid treatment effectively reduced CCL2 expression and CCR2^+^ monocyte recruitment. Further, plasma samples collected from individuals ascending from low to high altitudes showed increased thrombospondin-1 and TGF-β levels, which were reduced with dexamethasone. These findings reveal interstitial macrophage populations as potential therapeutic targets in hypoxic PH.

## Vascular remodeling in pulmonary hypertension

Pulmonary hypertension (PH) is a hemodynamic condition characterized by an elevation of the mean pulmonary arterial pressure (mPAP) greater than 20 mmHg, assessed by right heart catheterization (1). The 6th World Symposium on Pulmonary Hypertension (WSPH) in 2018 and the 2022 European Society of Cardiology and the European Respiratory Society (ESC/ERS) guidelines (2) offer a comprehensive classification for children and adults, divided into 5 subgroups, of which Group 1, pulmonary arterial hypertension (PAH) demonstrates the most severe pulmonary vascular remodeling compared with Group 2 PH, associated with left heart disease, and Group 3 PH, associated with lung disease and/or hypoxia. In general, progressive pulmonary vascular remodeling results in increased pulmonary vascular resistance and eventually right heart failure, which is why understanding the contributing factors to vascular remodeling is key for identifying therapeutic approaches.

The immune system plays an important role in the pathogenesis of PH, influencing both the development and the progression of the disease through complex immune responses (3). Peri- and intravascular inflammation is the hallmark of PH, and various immune cells, including macrophages, T cells, B cells, dendritic cells, and neutrophils, actively participate in the pulmonary vascular remodeling that characterizes PH. Macrophages can adopt different phenotypes in response to environmental cues, often leading to a predominance of proinflammatory macrophages that secrete chemokines, contributing to vascular inflammation and perivascular fibrosis (4). Neutrophils, involved in acute inflammatory processes, can infiltrate the pulmonary vasculature, releasing reactive oxygen species and proteolytic enzymes such as elastase, which further disrupt endothelial function, the extracellular matrix, and promote inflammation (5). T cells, particularly CD4^+^ Th17 cells activated by monocyte-derived DCs (6), have been implicated in driving inflammation and promoting vascular smooth muscle cell proliferation, whereas regulatory T cells (Tregs) seem to have a protective role (7). B cells contribute to the immune response in PH by producing autoantibodies and cytokines, exacerbating inflammation and vascular remodeling (8).

## The macrophage response in hypoxia-driven PH

While patients with Group 1 PAH develop severe vascular remodeling with neointima that often completely occludes small pulmonary arteries, PH due to hypoxia is characterized largely by medial hypertrophy and only milder degrees of neointima formation. Given the histological differences between Group 1 and Group 3 PH, it could be hypothesized that different players of the immune system contribute in distinct ways to subgroups of PH. This divergence puts into context the work of Kumar and colleagues in this issue of the JCI, who focused mainly on the role of macrophages, in particular interstitial macrophages, in Group 3 hypoxia-induced PH (9) as an example of clinical high-altitude PH.

Macrophages are widely distributed and have key roles in various physiologic and pathologic processes. Their diversity in function reflects their ability to respond to numerous signaling and environmental cues. While macrophages have traditionally been understood to polarize to either proinflammatory M1-like or antiinflammatory M2-like states, evidence has shown that they exist in a spectrum of states between those 2 phenotypic extremes (10). Several studies highlight the heterogeneity of lung interstitial macrophages and their response to hypoxia. Initially, proinflammatory interstitial macrophages accumulate in the perivascular space during early hypoxic exposure, exhibiting a PH program characterized by increased inflammatory gene activity and mitochondrial dysfunction. Over time, however, interstitial macrophage populations shift towards a more antiinflammatory and proreparative state, suggesting a dynamic adaptation in response to sustained hypoxic conditions (11). Macrophages contribute to vascular remodeling through various mechanisms, including the secretion of proinflammatory cytokines and factors that mediate smooth muscle cell proliferation and migration, thereby exacerbating vascular remodeling in PH. Antiinflammatory macrophage subsets, such as M2-regulatory macrophages, have shown potential in attenuating early pulmonary inflammation and mitigating vascular injury when introduced therapeutically (4). This duality underscores the importance of macrophages in both initiating inflammation and promoting repair processes within the vascular environment.

Kumar and authors studied hypoxia-induced pulmonary hypertension with hypoxia being the key driver in chronic lung disease as well as high-altitude PH (9). After exposing mice to hypoxia, they noted an increase in resident interstitial macrophages, which proliferated and expressed CCL2, a ligand for recruiting monocytes. Additionally, they noted a rise in recruited CCR2^+^ macrophages that expressed thrombospondin-1, a protein that activates TGF-β, which has been shown to contribute to vascular remodeling. Blocking monocyte recruitment through CCL2 neutralizing antibodies or CCR2 deficiency in the bone marrow partially reduced hypoxic PH. In the hypoxic mouse model, dexamethasone effectively reduced CCL2 expression and recruitment of CCR2^+^ monocytes. Similarly, human plasma samples collected from individuals ascending from low (225 m) to high (3500 m) altitudes showed increased thrombospondin-1 and TGF-β levels, which were reduced by antiinflammatory treatment with dexamethasone. The authors concluded that the pathological interaction between distinct interstitial macrophage populations may represent a therapeutic target for hypoxic PH.

## Considerations and conclusions

Importantly, further studies that explore alternative CCL2 sources and alternative stimuli beyond hypoxia that contribute to hypoxia-related PH are worth pursuing. The source of CCL2 production in response to hypoxia is most likely not restricted to interstitial macrophages of the lung. Hypoxia-stimulated CCL2 could very well be expressed by interstitial macrophages within other organs such as the brain and the intestine, and/or by other cell types such as endothelial cells, fibroblasts, smooth muscle cells, or adipocytes. While Kumar and colleagues have performed a multiorgan assessment of CCL2 expression in the heart, kidney, liver and spleen, CCL2 expression, in particular, in the intestine was not reported. The concept of a lung-gut axis in the context of hypoxia is explored in several studies (12). For instance, a review titled “The Bidirectional Gut-Lung Axis in Chronic Obstructive Pulmonary Disease” discusses how systemic hypoxia and oxidative stress in COPD may influence intestinal dysfunction, highlighting the role of the gut-lung axis in disease progression (13). Furthermore, blocking CCR2/CCL2 signaling only partially abrogated the PH phenotype, suggesting that parallel mechanisms, potentially involving other signaling pathways or other immune cells, contribute to hypoxic PH.

Other interesting questions and future directions include: (a) Addressing whether this paradigm of monocyte migration through CCL2/CCR2 signaling also plays a role in other forms of pulmonary hypertension, especially given that TGF-β–mediated vascular remodeling is known to be involved in Group 1 PAH. Additionally, therapeutic treatment with ActRIIA-Fc, a ligand trap for ligands of the TGF-β superfamily, has been shown to reverse proinflammatory and proliferative gene expression profiles and to normalize macrophage infiltration in diseased rodent lungs (14). Could ActRIIA-Fc also protect against hypoxia-induced PH, in mouse model and patients with Group 3 PH? (b) Recent single-cell RNA-seq (scRNA-seq) studies on lung tissue from patients with Scleroderma-associated PAH (SSc-PAH), idiopathic PAH (IPAH), and healthy donor controls using ligand-receptor signaling pairing revealed macrophage-to-endothelial cell signaling, especially in SSc-PAH compared to IPAH (15). Future scRNAseq studies in hypoxic PH could enhance our understanding of cell-cell interactions, helping to explain why certain macrophage populations accumulate in specific lung areas — primarily around pulmonary arteries as opposed to systemic arteries or pulmonary veins — and to which cells they signal. These studies could, therefore, uncover the signaling networks between various immune cells and vascular cells involved in the remodeling process with the goal to inhibit specific pathways for therapeutic purposes.

In conclusion, the interaction between different interstitial macrophage populations in the lung plays a crucial role in perivascular inflammation and vascular remodeling in hypoxia-induced PH. Understanding the macrophage dynamics and signaling pathways involved is important to develop effective treatment strategies for managing hypoxic PH, and the study by Kumar et al. presents a substantial step forward.

## Version 1 03/17/2025

Electronic publication
